# Germline DNA Repair Gene Mutations and Clonal Hematopoiesis (CH) in 24,849 Patients with BRCA-Associated Cancers

**DOI:** 10.3390/cancers17091432

**Published:** 2025-04-25

**Authors:** Catherine H. Marshall, Ali T. Arafa, Ellen Jaeger, Stamatina Fragkogianni, Anne Sonnenschein, Elizabeth Mauer, Lukasz P. Gondek, Calvin Chao, Jun Luo, Emmanuel S. Antonarakis

**Affiliations:** 1 Sidney Kimmel Comprehensive Cancer Center, Department of Oncology, Johns Hopkins School of Medicine, Baltimore, MD 21287, USA; chm@jhmi.edu (C.H.M.);; 2 Department of Pharmacology, University of Minnesota, Minneapolis, MN 55455, USA; 3 Tempus AI Inc., Chicago, IL 60654, USA; ellen.jaeger@tempus.com (E.J.); matina.fragkogianni@tempus.com (S.F.); elizabeth.mauer@tempus.com (E.M.);; 4 Department of Urology, James Buchanan Brady Urological Institute, Johns Hopkins School of Medicine, Baltimore, MD 21287, USA; 5 Department of Medicine, University of Minnesota Masonic Cancer Center, Minneapolis, MN 55455, USA

**Keywords:** clonal hematopoiesis, genetics, prostate cancer, breast cancer, ovarian cancer, pancreatic cancer, germline DNA repair defects

## Abstract

Purpose: To determine if the risk of clonal hematopoiesis (CH) would be higher among those with germline alterations in homologous recombination repair genes (gHRR) in the four BRCA-associated cancers (breast, ovarian, prostate, pancreas) compared to those without inherited predisposition (the sporadic group). Methods: We retrospectively analyzed deidentified data from 24,849 patient samples from the Tempus database with a primary diagnosis of breast, ovarian, prostate, and pancreatic cancers. Germline pathogenic or likely pathogenic variants in *BRCA1*, *BRCA2*, *ATM*, *PALB2*, and *CHEK2* were identified across all four cancer types. CH was determined based on the presence of pathogenic or likely pathogenic alterations in any one of 52 CH-associated genes with a variant allele fraction of at least 2% found in the normal match. Age-adjusted odds ratios were calculated for risk of CH across cancer types. Results: CH was identified in 14% of patients with BRCA-associated cancers. *DNMT3A*, *PPM1D*, and *TET2* were the most common CH gene alterations. After adjusting for age at time of biopsy, having any germline alteration in the breast cancer cohort was associated with a 41% increased likelihood of CH (OR 1.41; 95% CI 1.07–1.84, *p* = 0.014). An increase in CH prevalence was not seen in the three other cancer types. Conclusions: When accounting for age at time of testing, pathogenic germline alterations in DNA repair genes were associated with an increased risk of CH only among patients with breast cancer, but not in those with ovarian, pancreatic, or prostate cancers.

## Simple Summary

Clonal hematopoiesis (CH) is the expansion of white blood cells with mutations in genes associated with hematologic malignancies found in patients without evidence of any hematologic malignancy. CH is associated with increasing age and exposure to certain cancer therapies. We hypothesized that having a germline cancer predisposition gene mutation might be associated with an increased risk of CH. We used the deidentified Tempus dataset to answer this question. Among patients with breast cancer, we did see an association with increased risk of CH; this was not seen among those with prostate, ovarian, and pancreatic cancer.

## 1. Introduction

It is estimated that there were over 2 million new cancer diagnoses in the United States in 2024 and solid tumors make up the most common causes of cancer [1]. As cancer therapies improve and mortality rates decline, more consideration is needed of the potential comorbidities and risk factors patients may have that can contribute to morbidity related to cancer therapies [2]. Clonal hematopoiesis (CH) is one such risk factor that has been associated with a number of adverse outcomes including an increased risk of hematologic malignancy, cardiovascular disease, and death [3,4,5]. CH is common among patients with solid tumors [6]. Age is one of the strongest risk factors for CH, with the prevalence increasing in healthy patients with advancing age [3,7]. Smoking and certain prior cancer treatments, including alkylating chemotherapy, topoisomerase inhibitors, poly-ADP ribose polymerase (PARP) inhibitors, and radiation therapy, have also been associated with an increased risk of CH [8,9,10,11,12]. In the general population, the prevalence of CH ranges from about 10% in those age 70–79 to about 20% in those over 90 [7]. The prevalence of CH in patients with solid tumors seems to be higher with ranges between low estimates of about 14% and much higher estimates of around 65% depending on the tumor type, prior treatments, the detection sensitivity of the assay, and when in the course of disease CH is being investigated (treatment-naïve setting or after treatment for the primary malignancy) [6].

Less is known about the potential of inherited risk factors for CH. In the general population, genome-wide association studies have identified genes in the DNA repair pathway, such as *ATM*, as potentially contributing to an increased risk of CH [13]. Recently, germline mutations in a telomere-regulating gene, *POT1*, have also been linked to CH [14]. In an oncology population, there are less data. One previous study found that patients with germline *ATM* mutations have a higher risk of CH [15]. There are an increasing number of therapies being used that may increase the risk of CH and myeloid neoplasms among patients with cancer, and having a better understanding of pre-existing risk factors is needed [16]. Here, we used a large commercial clinical genomic database comprising DNA sequencing information from tumors and germlines to interrogate the prevalence of CH in germline-associated cancers and their sporadic counterparts. We hypothesized that the risk of CH would be higher among those with germline alterations in homologous recombination repair genes (gHRR) in the four BRCA-associated cancers (breast, ovarian, prostate, pancreas) compared to those without inherited predisposition (the sporadic group). 

## 2. Materials and Methods

### 2.1. Patients

We retrospectively analyzed deidentified data from 24,862 patient samples from the Tempus database with a primary diagnosis of breast, ovarian, prostate, and pancreatic cancers (termed BRCA-associated cancers) who underwent paired germline and somatic DNA sequencing with Tempus xT testing. Patients were stratified based on the presence or absence of germline alterations in one of five DNA repair genes of interest. Those germline genes of interest included *ATM*, *BRCA1*, *BRCA2*, *CHEK2*, and *PALB2*. In scenarios where patients had more than one tumor sample, only the most recently sequenced sample was used.

### 2.2. Sequencing Methods

Next-generation sequencing was conducted using the Tempus xT assay (Tempus AI, Inc., Chicago, IL, USA), as previously described [17,18]. Briefly, Tempus xT is a targeted, tumor–normal-matched DNA panel that detects single-nucleotide variants, insertions and/or deletions, and copy number variants in 648 genes, as well as chromosomal rearrangements in 22 genes with high sensitivity and specificity. Germline pathogenic or likely pathogenic variants in *BRCA1*, *BRCA2*, *ATM*, *PALB2*, and *CHEK2* were identified across all four cancer types from tumor–normal-matched analysis [19]. Patients with deleterious germline homologous recombination repair (HRR) variants were then separated in each cancer type from those that did not harbor any variants in these 5 HRR genes (termed the sporadic group). Classification of germline alterations was broadly based on recommendations from the American College of Medical Genetics (ACMG), the National Comprehensive Cancer Network (NCCN), and the published literature on inherited cancer syndromes [18]. CH was determined based on the presence of pathogenic or likely pathogenic alterations in any one of 52 CH-associated genes (Supplementary Table S1) with a variant allele fraction of at least 2% found in the normal match. This cut point was chosen as the clinical consequences of CH at variant allele frequencies of this level are the most commonly studied [4]. Pathogenicity is determined using Variants-Job, a proprietary tool to automate variant classification aligned to professional guidelines for variant curation. CH variant types included frameshift mutations; intron variants; splice donor, acceptor, and region variants; stop gains and losses; synonymous variants; disruptive in-frame deletions and insertions; and missense variants. These variants were distinguished from potential germline variants and sequencing artifacts based on features including number of supporting reads for the alternate allele, variant type and length, relative variant allele fraction in the tumor and normal match, and estimated copy number in the tumor.

### 2.3. Statistical Methods

Patients with germline alterations in *BRCA1*, *BRCA2*, *ATM*, *CHEK2*, and *PALB2* were compared to those without germline HRR alterations (sporadic). Those patients with more than one germline alteration of interest were excluded given we were trying to understand the individual contributions of each germline genetic alteration and did not want to confound the results by combining patients with multiple concurrent germline alterations. Statistical comparisons were conducted between all germline groups using the Kruskal–Wallis rank sum test and Pearson’s chi-squared test for continuous variables (age at biopsy) and categorical variables (sex, race, ethnicity, and stage of disease), respectively. Dunn’s post hoc test was performed to assess which specific germline groups had significant differences in age at biopsy, with false discovery rate correction for multiple testing. Associations between age at biopsy, presence of germline mutations, specific germline mutations, sex, and prior radiation exposure on CH prevalence were assessed using Firth’s logistic regression, with confidence intervals calculated by penalized profile likelihood-based methods. Analyses were only performed on groups with size > 10. Statistical significance was established at an alpha level of 0.05, indicating that differences with a *p*-value below this threshold were considered significant.

## 3. Results

**Figure 1:**
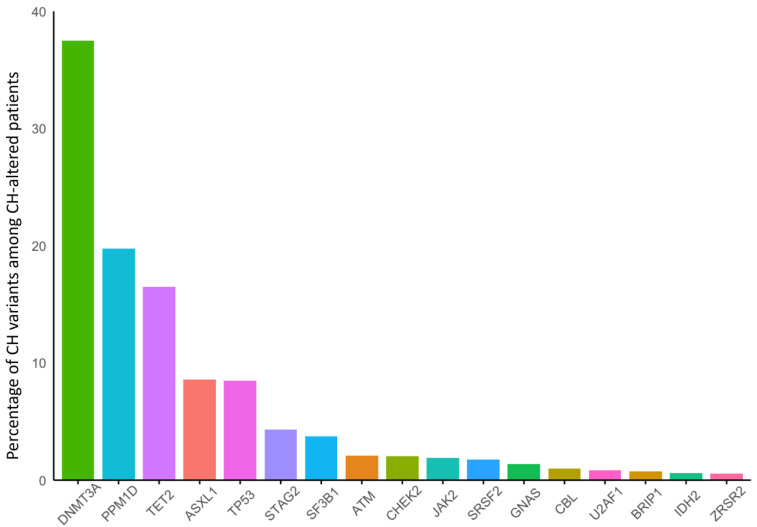
Distribution of clonal hematopoiesis (CH) mutations occurring in ≥1% of patients with CH overall.

Overall, 24,849 patients were included after 13 patients were excluded for having multiple germline mutations in genes of interest. CH was identified in 3548 (14%) of 24,849 patients. Most patients (2879/3548, 81%) had one pathogenic CH variant identified, while 530 (15%) had two and 139 (4%) had more than two pathogenic CH variants. The most common CH variants found were in *DNMT3A* (N = 1330, 37%), followed by *PPM1D* (N = 700, 20%), *TET2* (N = 585, 16%), *TP53* (N = 301, 8.5%), and *ASXL1* (N = 304, 8.6%) (Figure 1). The presence of CH was associated with increasing age at time of biopsy (odds ratio (OR) 1.05 per year of increasing age, 95% CI 1.05–1.06, *p* < 0.001).

### 3.1. Breast Cancer

**Figure 2:**
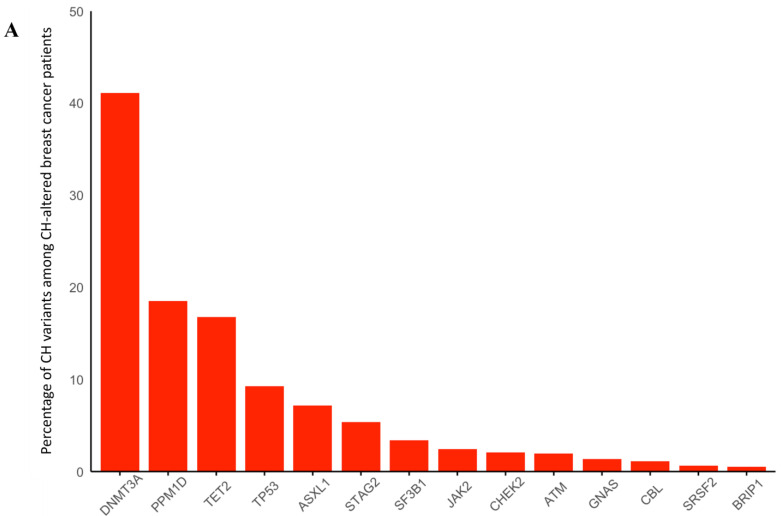

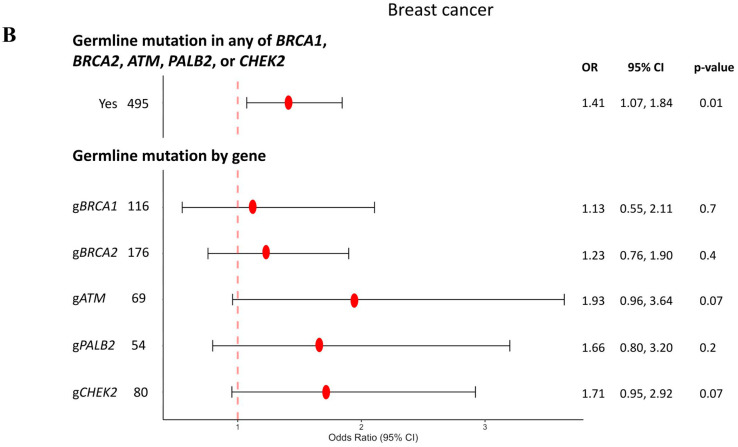
(**A**) Distribution of clonal hematopoiesis (CH) mutations occurring in ≥1% of patients with breast cancer and CH. (**B**) combined forest plots for age-adjusted odds of having CH with having any germline alteration in *BRCA1*, *BRCA2*, *ATM*, *PALB2*, or *CHEK2*, and by specific gene (compared to the sporadic counterparts).

In 8086 patients with breast cancer, there were 7591 patients without germline alterations, 116 patients (1.4%) with germline *BRCA1* alterations, 176 (2.2%) with germline *BRCA2* alterations, 69 (0.9%) with germline *ATM* alterations, 54 (0.7%) with germline *PALB2* alterations, and 80 (1.0%) with germline *CHEK2* alterations. The median age at sample collection was different across groups, with the lowest among those with germline *BRCA1* alterations (median 48, IQR 38–59) and those with germline *CHEK2* with the oldest median age of 63 (IQR 55–70, *p* < 0.001) (Table 1). Overall, 12% of the patients had CH, which ranged from 9% of those with germline *BRCA1* alterations to 22% among those with germline *PALB2* alterations. The most common CH alterations were in *DNMT3A* followed by *PPM1D*, *TET2*, and *TP53* (Figure 2A). The presence of CH was associated with increasing age (OR 1.05 per year; 95% CI 1.05–1.06, *p* < 0.001) and prior radiation exposure (OR 1.20, 95% CI 1.01–1.41, *p* = 0.034). After adjusting for age at time of biopsy, we found an association between having any germline alteration and an increased likelihood of CH (OR 1.41; 95% CI 1.07–1.84, *p* = 0.014); however, there was no significant increase in CH among any of the patient populations with individual germline alterations (Figure 2B).

### 3.2. Ovarian Cancer

In 5018 patients with ovarian cancer, there were 4689 patients without germline alterations, 175 patients (3.5%) with germline *BRCA1* alterations, 102 (2.0%) with germline *BRCA2* alterations, 27 (0.5%) with germline *ATM* alterations, 12 (0.2%) with germline *PALB2* alterations, and 13 (0.3%) with germline *CHEK2* alterations. The median age at sample collection was lowest among those with *BRCA1* alterations (56, IQR 49–64) compared to those with germline *PALB2* mutations, which had the oldest median age of 70 (IQR 64–73, *p* < 0.001 for group difference) (Table 2).

**Figure 3:**
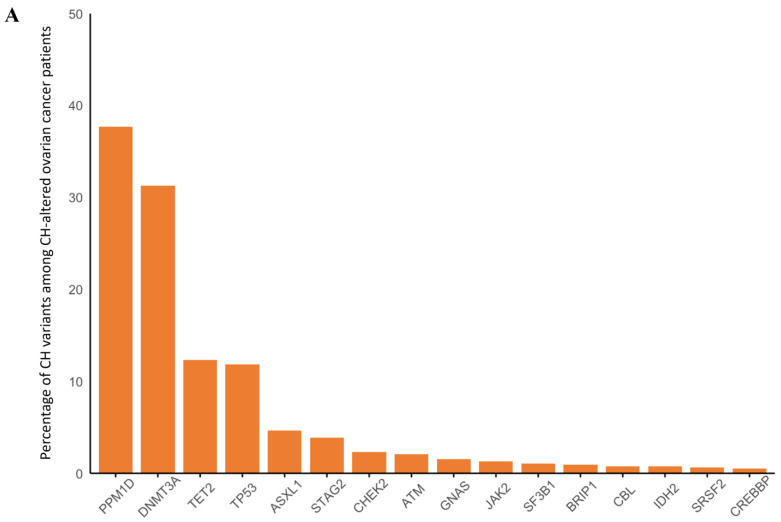

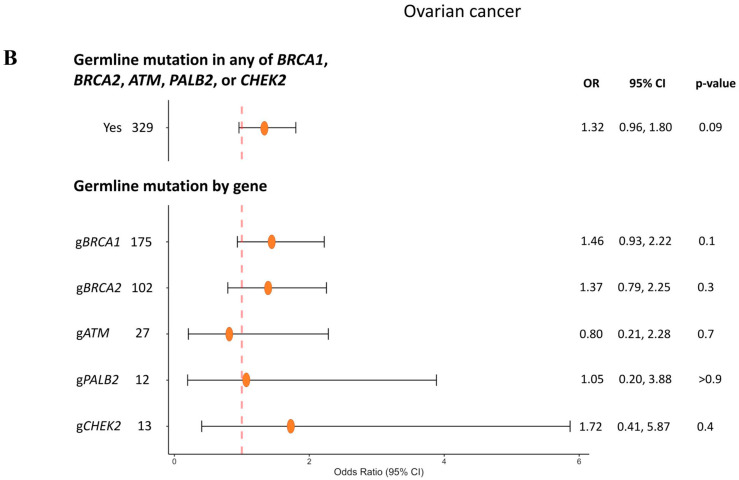
(**A**) Distribution of clonal hematopoiesis (CH) mutations occurring in ≥1% of patients with ovarian cancer and CH. (**B**) combined forest plots for age-adjusted odds of having CH with having any germline alteration in *BRCA1*, *BRCA2*, *ATM*, *PALB2*, or *CHEK2*, and by specific gene (compared to the sporadic counterparts).

Overall, 15% of the patients had CH which ranged from 11% of those with germline *ATM* alterations to 23% among those with germline *CHEK2* alterations. The most common alterations overall were in *PPM1D*, *DNMT3A*, *TET2*, *TP53*, and *ASXL1* (Figure 3A). The presence of CH was associated with increasing age (OR 1.05 per year; 95% CI 1.04–1.06, *p* < 0.001). There was no association between having any germline alteration and the likelihood of CH and there was no significant increase in CH among any of the patient populations with germline alterations (Figure 3B) after adjusting for age at biopsy.

### 3.3. Prostate Cancer

**Figure 4:**
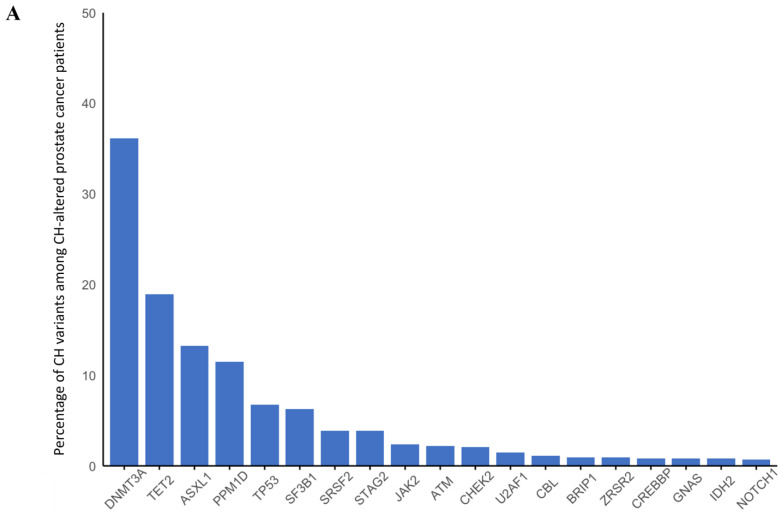

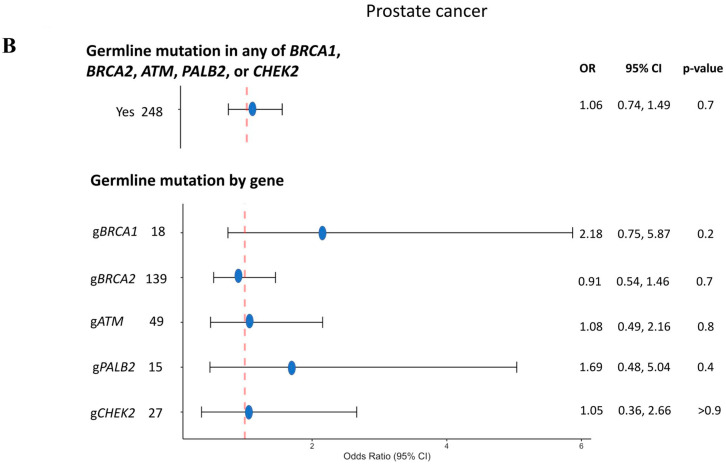
(**A**) Distribution of clonal hematopoiesis (CH) mutations occurring in ≥1% of patients with prostate cancer and CH. (**B**) combined forest plots for age-adjusted odds of having CH with having any germline alteration in *BRCA1*, *BRCA2*, *ATM*, *PALB2*, or *CHEK2*, and by specific gene (compared to the sporadic counterparts).

There were 5271 prostate cancer patients included with 18 (0.3%) having germline *BRCA1* alterations, 139 (2.6%) with *BRCA2* alterations, 49 (0.9%) with germline *ATM* alterations, 15 (0.3%) with germline *PALB2* alterations, and 27 (0.5%) with germline *CHEK2* alterations. Median age was significantly different across the group; it was the highest among those with germline *CHEK2* alterations (72 years; IQR 66–79) and the lowest among those with germline *BRCA2* alterations (65 years; IQR 60–70 years, *p* = 0.002) (Table 3). CH was present in 17% of the patients overall with *DNMT3A* being the most common gene followed by *TET2*, *ASXL1*, and then *PPM1D* alterations (Figure 4A). CH was associated with increasing age (OR 1.06 per year; 95% CI 1.05–1.07; *p* < 0.001) and prior radiation treatment (OR 1.36; 95% CI 1.06–1.73; *p* = 0.016). Germline alterations were not associated with an increased risk of CH after adjusting for age at biopsy (Figure 4B).

### 3.4. Pancreatic Cancer

**Figure 5:**
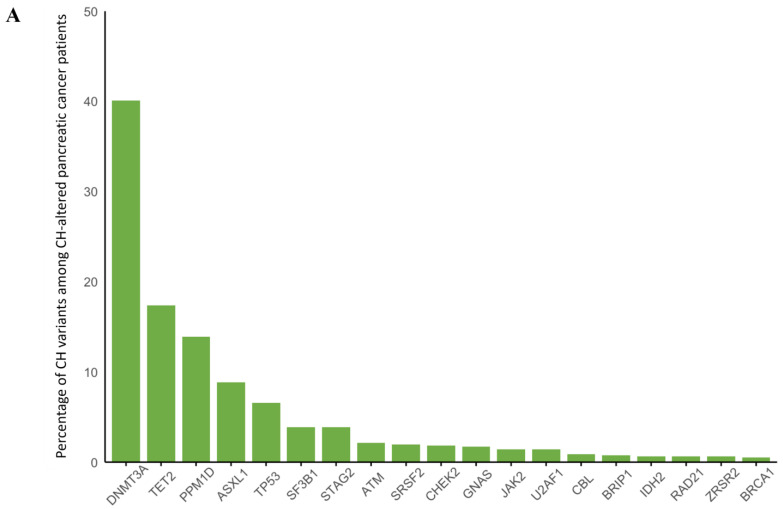

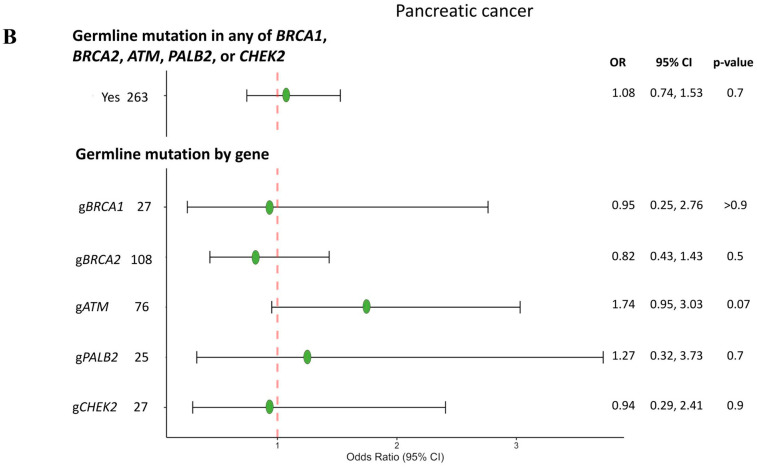
(**A**) Distribution of clonal hematopoiesis (CH) mutations occurring in ≥1% of patients with pancreatic cancer and CH. (**B**) combined forest plots for age-adjusted odds of having CH with having any germline alteration in *BRCA1*, *BRCA2*, *ATM*, *PALB2*, or *CHEK2*, and by specific gene (compared to the sporadic counterparts).

Of the 6474 patients with pancreatic cancer, 27 (0.4%) had *BRCA1* alterations, 108 (1.7%) had *BRCA2* alterations, 76 (1.2%) had germline *ATM* alterations, 25 (0.4%) had *PALB2* alterations, and 27 (0.4%) had *CHEK2* alterations. Median age ranged from 62 (IQR 54–71) among those with germline *BRCA1* alterations to 68 (IQR 62–74) among those with germline *CHEK2* alterations (*p* = 0.002 for group comparison) (Table 4). Overall, 14% of patients had CH, with the age-related CH genes (*DNMT3A*, *TET2*, and *ASXL1*) predominating (Figure 5A). CH was associated with older age (OR 1.06 per year, 95% CI 1.05–1.07, *p* < 0.001) and prior radiation exposure (OR 1.40, 95% CI 1.04–1.84, *p* = 0.025) but was not associated with the presence of a germline alteration after adjusting for age at biopsy (Figure 5B).

## 4. Discussion

In this study of patients with BRCA-associated solid tumors (breast, ovarian, pancreatic, and prostate cancers), we found that clonal hematopoiesis (CH) was common and was associated with advancing age and prior radiation exposure, as has been found in other studies [7,11,20]. The prevalence of CH is similar to what has been found in other studies of patients with prostate cancer [6]. Patients with germline DNA repair alterations in some, but not all, cancers have younger ages of onset [21,22]. Therefore, this may confound subsequent studies exploring a potential link between germline alterations in DNA repair genes and CH among patients who have already developed cancer. The novel finding in our study is that after controlling for age, which is necessary when considering the potential differences in age of onset of inherited malignancies, the presence of a germline alteration in any of the five DNA repair genes studied (*ATM*, *BRCA1/2*, *CHEK2*, *PALB2*) was not associated with a higher prevalence of CH, except in aggregate among women with breast cancer. This suggests that there may be differences by cancer type, and that the risk of CH among patients with cancer needs to be explored not only in aggregate but also by subtype of cancer. Other prior studies have found germline *ATM* alterations to contribute to an increased risk of CH, which we did not observe in our study [13,15]. A possible reason for this is that our sample size was smaller and we limited our sample size to enrich the population with BRCA-associated cancers. Another consideration is that our study really is reflective of treatment-related CH, which develops after selective pressure imposed by cancer therapies and is distinct from age-related CH [23]. Therapy-related CH includes mutations in *PPM1D* and *TP53* which are almost always seen as a result of selective pressures from previous cancer treatment [24,25]. This is evident in our study as *PPM1D* alterations were the most common CH alterations among patients with ovarian cancer and the second most common CH alteration among patients with breast cancer. *TP53* mutations were also the fourth or fifth most common alteration found in the cohort overall. This likely reflects differences in exposure to therapies, such as PARP inhibitors, which are associated with the development of CH and are widely used in breast and ovarian cancer but less so in pancreas and prostate cancers [26,27,28]. Future research will be needed to determine how CH develops longitudinally in various at-risk populations of patients with cancer, and how it changes after exposure to cancer therapies among patients with inherited germline predispositions as there is some early evidence that this process varies and may even be blunted among patients with germline DNA repair alterations [29]. Germline genetic alterations are being used to personalize therapeutic choices for patients, and the long-term implications of how these therapies interact with germline predispositions and the development of CH remain an active area of research. A more thorough understanding of how CH interacts with tumors and the tumor microenvironment is also needed as early research suggests an impact on the microenvironment [30].

There are several limitations of our study. While our study included nearly 25,000 patients overall, the number of patients with germline alterations is still relatively small by comparison so we cannot completely rule out an association of small magnitude between a particular germline HRR mutation and CH prevalence. However, if such an association exists, the effect size is likely small. Additionally, in considering studies of effects of germline alterations on CH among patients with cancer, age is a significant confounder, not only influencing the age of onset of the cancer itself, but also potentially influencing the onset of specific CH clones, for example, with *ATM* clonal hematopoiesis mutations appearing earlier than others [13]. We did not explore multiple samples from patients and only considered the most recent sample. This was because CH is associated with age and we wanted to choose the latest sample to determine if there was a signal. Finally, there are potential limitations in germline genetic sequencing [31]. As liquid biopsies are increasingly being used in clinical practice, the identification of CH will undoubtedly increase and most significantly, can potentially interfere with the interpretation of actionable mutations [32,33].

## 5. Conclusions

Common CH variants are found across patients with BRCA-associated tumor types, with an overall CH prevalence of 14% in these cancers. When accounting for age at time of testing, pathogenic germline alterations in DNA repair genes were associated with an increased risk of CH only among those with breast, but not in patients with ovarian, pancreatic, or prostate cancer. The clinical implications of CH detection in BRCA-associated cancers requires further exploration.

## Figures and Tables

**Table 1:** Breast cancer cohort demographics.

	Sporadic	g*BRCA1*	g*BRCA2*	g*ATM*	g*PALB2*	g*CHEK2*
N (%)	7591 (94)	116 (1)	176 (2)	69 (1)	54 (1)	80 (1)
Age at testing, mean	60	48	56	59	58	63
IQR	50, 69	38, 59	45, 64	44, 69	50, 74	55, 70
N (%) Female	7511 (99)	116 (100)	164 (93)	68 (99)	54 (100)	78 (98)
Race, N (%)						
White	3761 (50)	46 (40)	72 (41)	35 (51)	26 (48)	45 (56)
Black	725 (10)	9 (8)	19 (11)	4 (6)	3 (6)	1 (1)
Asian	232 (3)	5 (4)	7 (4)	2 (3)	3 (6)	1 (1)
Other	409 (5)	8 (7)	14 (8)	1 (1)	5 (9)	5 (6)
Hispanic, N (%)	489 (6)	9 (8)	17 (10)	4 (6)	6 (11)	6 (8)
Stage						
I	127 (2)	0 (0)	2 (1)	0 (0)	1 (2)	0 (0)
II	309 (4)	5 (4)	9 (5)	3 (4)	6 (11)	2 (3)
III	333 (4)	5 (4)	9 (5)	4 (6)	0 (0)	1 (1)
IV	3973 (52)	49 (42)	87 (49)	39 (57)	30 (56)	52 (65)
N (%) with CH	899 (12)	10 (9)	23 (13)	12 (17)	12 (22)	17 (21)

**Table 2:** Ovarian cancer cohort demographics.

	Sporadic	g*BRCA1*	g*BRCA2*	g*ATM*	g*PALB2*	g*CHEK2*
N (%)	4689 (93)	175 (4)	102 (2)	27 (1)	12 (<1)	13 (<1)
Age at testing, median	65	56	64	63	70	58
IQR	56, 72	49, 64	57, 69	54, 71	64, 73	45, 70
Race, N (%)						
White	2576 (55)	93 (53)	51 (50)	14 (52)	10 (83)	7 (54)
Black	260 (6)	8 (5)	5 (5)	2 (7)	0 (0)	0 (0)
Asian	124 (3)	6 (3)	5 (5)	1 (4)	0 (0)	0 (0)
Other	199 (4)	10 (6)	2 (2)	1 (4)	0 (0)	0 (0)
Hispanic, N (%)	258 (6)	15 (9)	2 (2)	0 (0)	0 (0)	0 (0)
Stage						
I	87 (2)	1 (1)	0 (0)	0 (0)	0 (0)	0 (0)
II	96 (2)	2 (1)	1 (1)	0 (0)	1 (8)	0 (0)
III	647 (14)	14 (8)	10 (10)	1 (4)	1 (8)	2 (15)
IV	855 (18)	31 (18)	16 (16)	5 (19)	2 (17)	4 (31)
N (%) with CH	715 (15)	28 (16)	19 (19)	3 (11)	2 (17)	3 (23)

**Table 3:** Prostate cancer cohort demographics.

	Sporadic	g*BRCA1*	g*BRCA2*	g*ATM*	g*PALB2*	g*CHEK2*
N (%)	5023 (95)	18 (<1)	139 (3)	49 (1)	15 (<1)	27 (1)
Age at testing, median	66	65	63	66	69	70
IQR	61, 74	60, 78	60, 70	62, 74	63, 73	66, 79
Race, N (%)						
White	2366 (47)	9 (50)	74 (53)	24 (49)	10 (67)	16 (59)
Black	555 (11)	0 (0)	16 (12)	4 (8)	0 (0)	0 (0)
Asian	89 (2)	2 (11)	0 (0)	1 (2)	0 (0)	0 (0)
Other	158 (3)	0 (0)	4 (3)	1 (2)	1 (7)	0 (0)
Hispanic, N (%)	239 (5)	1 (6)	10 (7)	3 (6)	0 (0)	0 (0)
Stage						
I	8 (<1)	0 (0)	0 (0)	0 (0)	0 (0)	0 (0)
II	64 (1)	1 (6)	0 (0)	0 (0)	0 (0)	0 (0)
III	193 (4)	1 (6)	4 (3)	1 (2)	0 (0)	1 (4)
IV	2487 (50)	4 (22)	75 (54)	24 (49)	10 (67)	21 (78)
N (%) with CH	834 (17)	6 (33)	19 (14)	9 (18)	4 (27)	5 (19)

**Table 4:** Pancreas cancer cohort demographics.

	Sporadic	g*BRCA1*	g*BRCA2*	g*ATM*	g*PALB2*	g*CHEK2*
N (%)	6211 (96)	27 (<1)	108 (2)	76 (1)	25 (<1)	27 (<1)
Age at testing, median	67	62	65	65	64	68
IQR	60, 74	54, 71	58, 72	61, 72	55, 67	62, 74
N (%) Female	2898 (47)	11 (41)	48 (44)	37 (49)	14 (56)	11 (41)
Race, N (%)						
White	3071 (49)	10 (37)	50 (46)	30 (39)	22 (88)	19 (70)
Black	393 (6)	3 (11)	4 (4)	6 (8)	0 (0)	0 (0)
Asian	132 (2)	0 (0)	6 (6)	1 (1)	0 (0)	0 (0)
Other	164 (3)	2 (7)	4 (4)	4 (5)	0 (0)	0 (0)
Hispanic, N (%)	243 (4)	1 (4)	8 (7)	5 (7)	0 (0)	0 (0)
Stage						
I	203 (3)	2 (7)	6 (6)	2 (3)	0 (0)	1 (4)
II	416 (7)	2 (7)	5 (5)	9 (12)	1 (4)	3 (11)
III	321 (5)	1 (4)	3 (3)	6 (8)	2 (8)	2 (7)
IV	3372 (54)	15 (56)	73 (68)	28 (37)	15 (60)	16 (59)
N (%) with CH	889 (14)	3 (11)	13 (12)	16 (21)	3 (12)	4 (15)

## Data Availability

Deidentified data used in the research was collected in a real-world health care setting and is subject to controlled access for privacy and proprietary reasons. When possible, derived data supporting the findings of this study have been made available within the paper and its Supplementary Table S1.

## Supplementary Materials

The following supporting information can be downloaded at: https://www.mdpi.com/article/10.3390/cancers17091432/s1, Table S1: 52 CH-associated genes with alterations identified.

## Abbreviations

The following abbreviations are used in this manuscript:

CH	Clonal hematopoiesis
gHRR	Germline Homologous recombination repair
HRR	Homologous recombination repair
IQR	Interquartile range
OR	Odds ratio
PARP	poly-ADP ribose polymerase
